# Supplementary material for: Targeted sequencing of candidate genes of dyslipidemia in Punjabi Sikhs: Population-specific rare variants in GCKR promote ectopic fat deposition
Source: PLoS One. 2019 Aug 1;14(8):e0211661. doi: 10.1371/journal.pone.0211661 (PMC6675050; doi:10.1371/journal.pone.0211661)
Supplement: S1 Fig — Flow Chart summarizes research design and targeted sequencing and replication and functional studies workflow. (PDF) [file pone.0211661.s001.pdf]

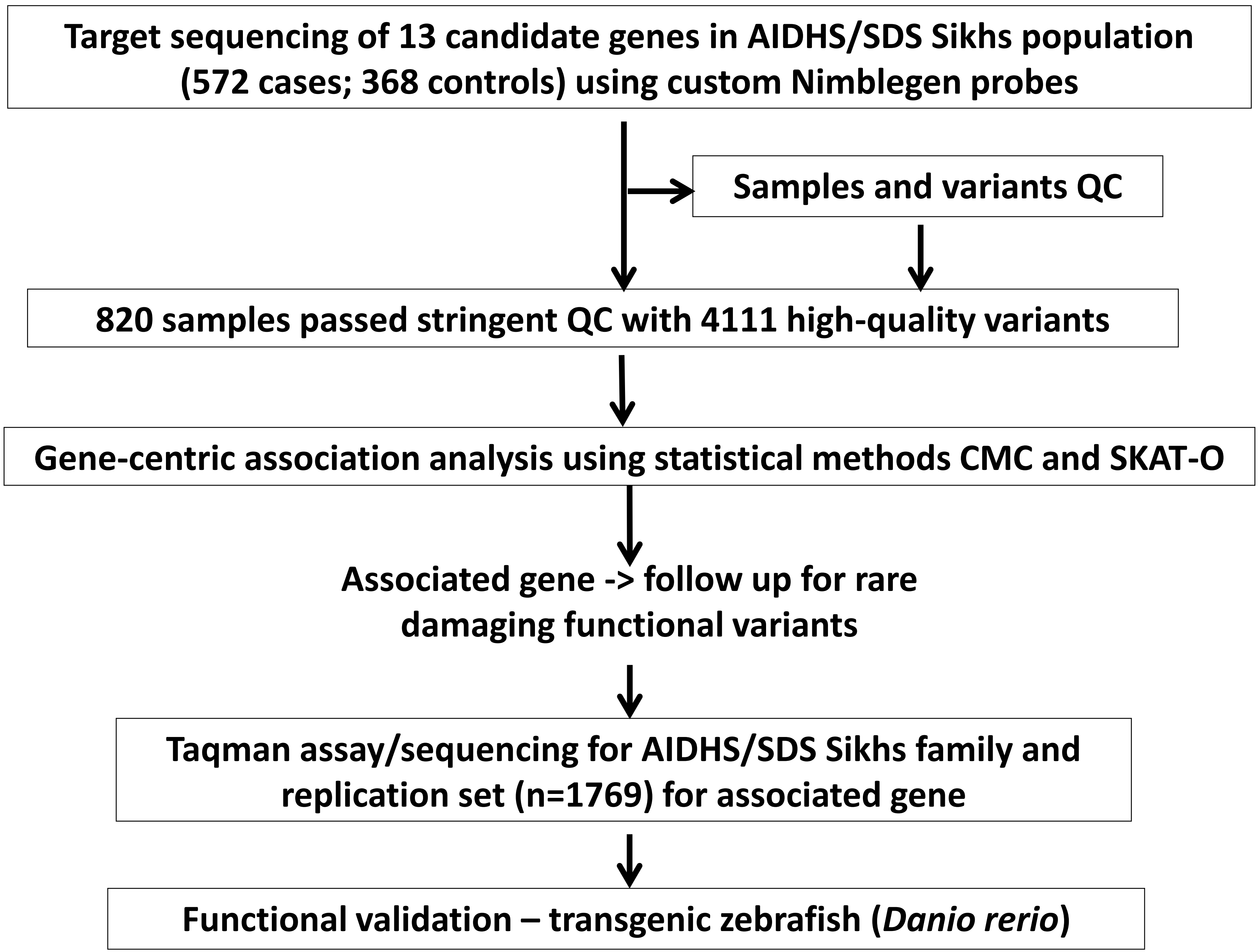
Target sequencing of 13 candidate genes in AIDHS/SDS Sikhs population (572 cases; 368 controls) using custom Nimblegen probes
Samples and variants QC
820 samples passed stringent QC with 4111 high-quality variants
Gene-centric association analysis using statistical methods CMC and SKAT-O
Associated gene -> follow up for rare damaging functional variants
Taqman assay/sequencing for AIDHS/SDS Sikhs family and replication set (n=1769) for associated gene
Functional validation – transgenic zebrafish (*Danio rerio*)
