# Supplementary figures and images for: Targeted sequencing of candidate genes of dyslipidemia in Punjabi Sikhs: Population-specific rare variants in GCKR promote ectopic fat deposition
Source: PLoS One. 2019 Aug 1;14(8):e0211661. doi: 10.1371/journal.pone.0211661 (PMC6675050; doi:10.1371/journal.pone.0211661)

Figure 2S A-B


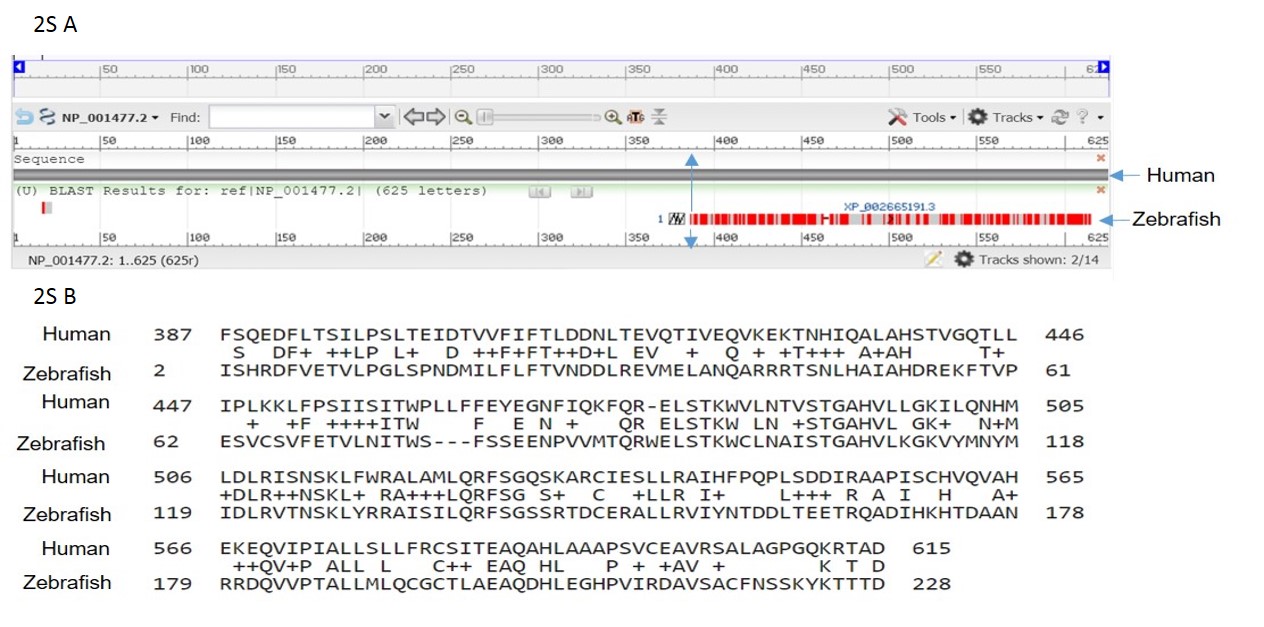

Supplement: S2 Fig — A. Upper gray horizontal bar is the protein sequence is human GCKR (NP_001477.2) with 625 amino acids and lower red horizontal block is the zebrafish GCKR (XP_002665191.3) protein sequence. S2 Fig B. Protein sequence alignment of human and zebrafish GCKR. Upper rows represent human residues and lower rows represent zebrafish residues starting at codon 387 of human GCKR. The black spaces and + symbols indicate low degree of homolog between human and zebrafish, only 94 out of 233 (41%) residues showed complete alignment. (DOCX) [file pone.0211661.s002.docx]

**Normal Diet High Fat Diet**


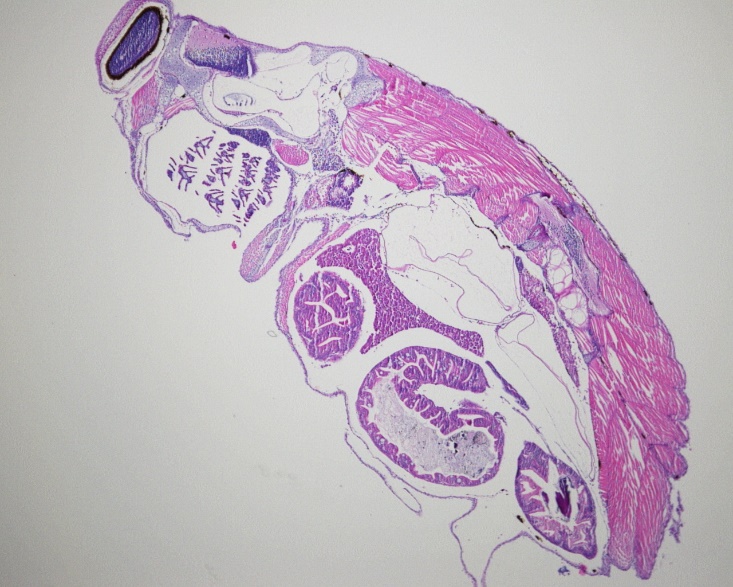

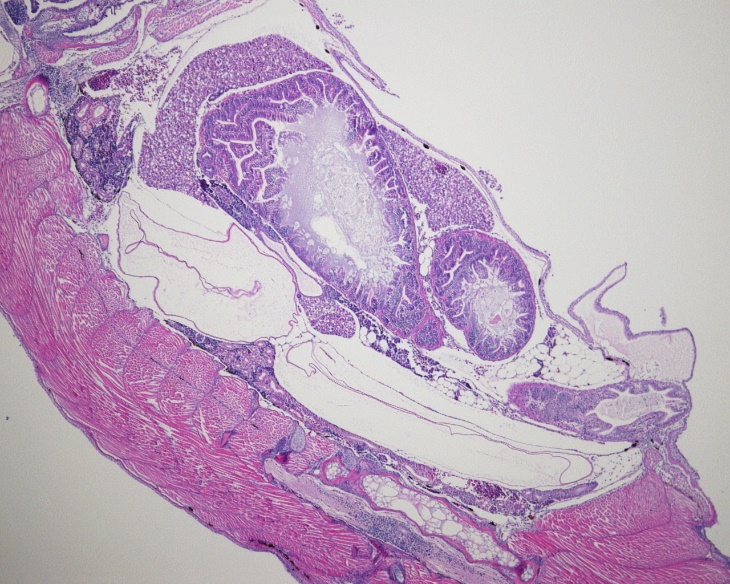


**Wild-Type TAB 5 (3S-A)**


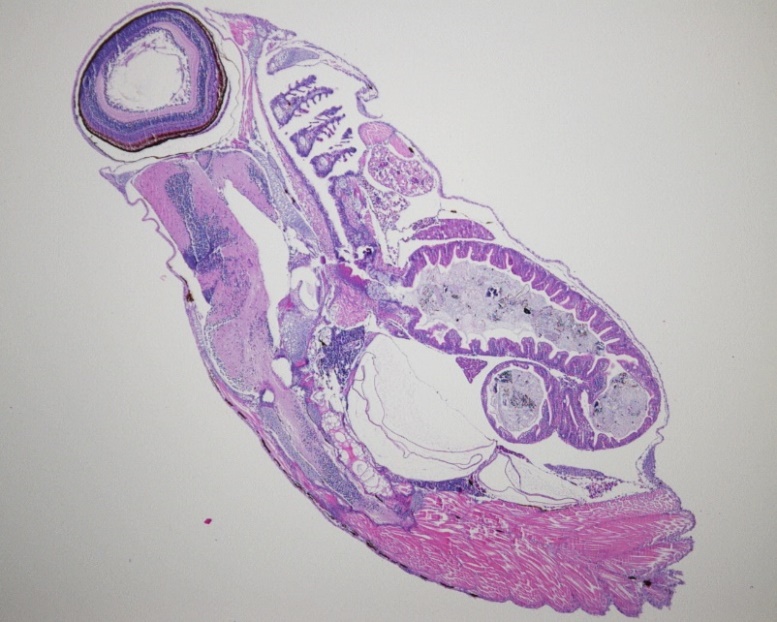

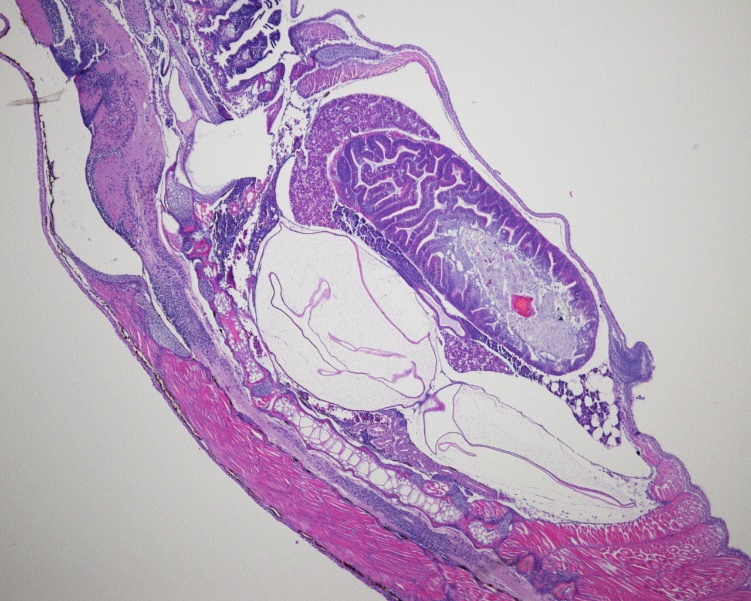


**Transgenic normal *GCKR* (3S-B)**


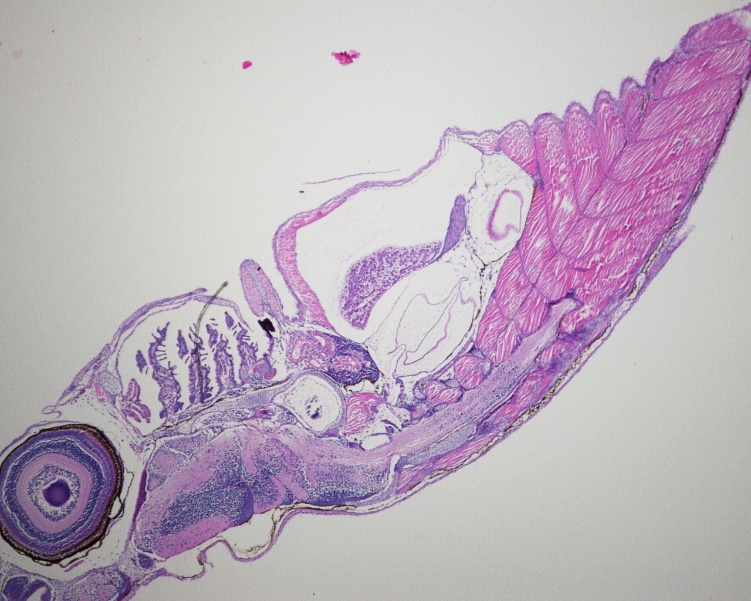

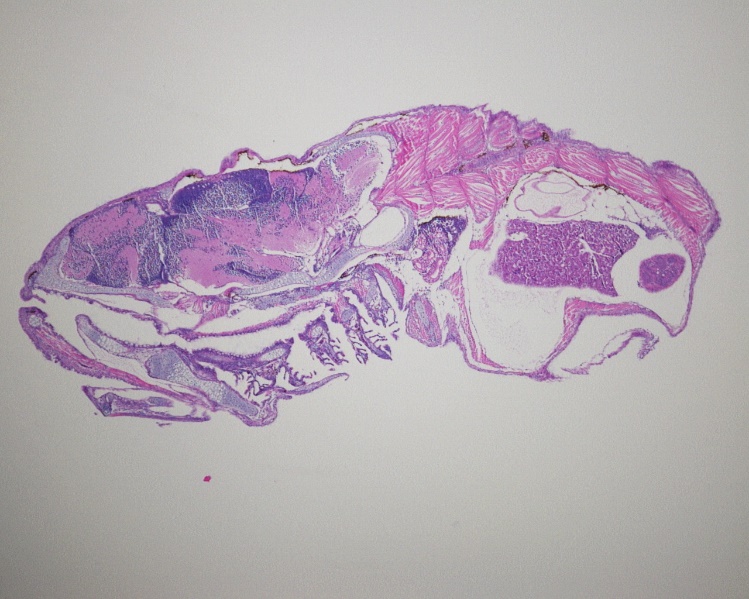


**Transgenic mutant *GCKR* (3S-C)**

Supplement: S3 Fig — General observation of zebrafish larvae from three groups fed on a normal and high fat diet at 4X magnification. (DOCX) [file pone.0211661.s003.docx]
