# Supplementary material for: Targeted sequencing of candidate genes of dyslipidemia in Punjabi Sikhs: Population-specific rare variants in GCKR promote ectopic fat deposition
Source: PLoS One. 2019 Aug 1;14(8):e0211661. doi: 10.1371/journal.pone.0211661 (PMC6675050; doi:10.1371/journal.pone.0211661)
Supplement: S1 Table — (DOCX) [file pone.0211661.s004.docx]

Table 1S: Gene-centric association of coding variants using combined multivariate and collapsing (CMC) and SKAT-O (uniform) analyses.

| Chr | Position | Gene name | CMC *p* value | SKAT-O *p* value |
| --- | --- | --- | --- | --- |
| 1 | 62915397 | *DOCK7* | 0.912 | 0.880 |
| 1 | 63058158 | *ANGPTL3* | 0.912 | 0.880 |
| 2 | 27714706 | ***GCKR*** | **2.1x10^-05^** | **8.1x10^-05^** |
| 7 | 73002524 | ***MLXIPL*** | **0.067** | **1.6x10^-02^** |
| 8 | 19791582 | ***LPL*** | **1.06x10^-03^** | **0.051** |
| 8 | 126437563 | *TRIB1* | 0.961 | 0.753 |
| 11 | 116613886 | *BUD13* | 0.054 | 0.401 |
| 11 | 116644276 | *ZNF259* | 0.683 | 0.674 |
| 11 | 116655086 | *APOA5* | 0.745 | 0.673 |
| 11 | 116695624 | *APOC3* | 0.228 | 3.1x10^-02^ |
| 11 | 116701469 | *APOA1* | 0.495 | 0.112 |
| 19 | 45404039 | *APOE* | 0.817 | 0.575 |
| 19 | 45412921 | *APOC1* | 0.129 | 0.104 |
