# Supplementary material for: Targeted sequencing of candidate genes of dyslipidemia in Punjabi Sikhs: Population-specific rare variants in GCKR promote ectopic fat deposition
Source: PLoS One. 2019 Aug 1;14(8):e0211661. doi: 10.1371/journal.pone.0211661 (PMC6675050; doi:10.1371/journal.pone.0211661)
Supplement: S2 Table — (DOCX) [file pone.0211661.s005.docx]

Table 2S: Summary of classification and distribution of high quality variants identified in targeted sequencing of 13 gene regions including GCKR, LPL, and MLXIPL gene regions

| Gene | Variant classification | Total variants | Case (n=572) | Controls (n=248) |
| --- | --- | --- | --- | --- |
|  | Coding | 268 | 211 | 133 |
|  | Nonsyn/stop/splicing/Indel | 187 | 145 | 87 |
| Combined | Synonymous | 81 | 66 | 46 |
|  | UTR Region | 250 | 211 | 123 |
|  | Intergenic | 1102 | 973 | 545 |
|  | Intronic | 2491 | 2183 | 1276 |
|  | Total | 4111 | 3578 | 2077 |
|  | Coding | 27 | 24 | 11 |
|  | Nonsyn/stop/splicing/Indel | 21 | 20 | 7 |
| GCKR | Synonymous | 6 | 4 | 4 |
|  | UTR Region | 2 | 1 | 1 |
|  | Intergenic | 0 | 0 | 0 |
|  | Intronic | 420 | 362 | 194 |
|  | Total | 449 | 411 | 217 |
|  | Coding | 28 | 24 | 15 |
|  | Nonsyn/stop/splicing/Indel | 21 | 17 | 10 |
| LPL | Synonymous | 7 | 7 | 5 |
|  | UTR Region | 45 | 39 | 28 |
|  | Intergenic | 0 | 0 | 0 |
|  | Intronic | 464 | 412 | 253 |
|  | Total | 537 | 499 | 311 |
|  | Coding | 56 | 47 | 25 |
|  | Nonsyn/stop/splicing/Indel | 32 | 25 | 15 |
| MLXIPL | Synonymous | 24 | 22 | 10 |
|  | UTR Region | 35 | 32 | 15 |
|  | Intergenic | 0 | 0 | 0 |
|  | Intronic | 582 | 508 | 277 |
|  | Total | 673 | 634 | 342 |

Table 2S: Summary of classification and distribution of 4111 high quality variants identified in the discovery cohort using targeted sequencing.

| Gene | Variant classification | Total variants | Case (n=572) | Controls (n=248) |
| --- | --- | --- | --- | --- |
|  | Coding | 268 | 211 | 133 |
|  | Nonsyn/stop/splicing/Indel | 187 | 145 | 87 |
| Combined | Synonymous | 81 | 66 | 46 |
|  | UTR Region | 250 | 211 | 123 |
|  | Intergenic | 1102 | 973 | 545 |
|  | Intronic | 2491 | 2183 | 1276 |
|  | Total | 4111 | 3578 | 2077 |

| Gene | Variant classification | Total variants | Case (n=572) | Controls (n=248) |
| --- | --- | --- | --- | --- |
|  | Coding | 175 | 147 | 96 |
|  | Nonsyn/stop/splicing/Indel | 115 | 95 | 61 |
| Combined | Synonymous | 60 | 52 | 35 |
|  | Upstream/downstream | 290 | 253 | 167 |
|  | Intergenic | 485 | 416 | 296 |
|  | Intronic | 1411 | 1224 | 849 |
|  | Total | 2361 | 2040 | 1408 |
