# Supplementary material for: Targeted sequencing of candidate genes of dyslipidemia in Punjabi Sikhs: Population-specific rare variants in GCKR promote ectopic fat deposition
Source: PLoS One. 2019 Aug 1;14(8):e0211661. doi: 10.1371/journal.pone.0211661 (PMC6675050; doi:10.1371/journal.pone.0211661)
Supplement: S3 Table — A-C. (S3 Table-A). Demographic and clinical traits of carriers of GCKR S105N (rs774930016) variant in subjects from the AIDHS (S3 Table-B). Demographic and clinical traits of carriers of GCKR R297Q (rs760427565) variant in subjects from the AIDHS. (S3 Table-C). Demographic and clinical traits of carriers of GCKR R553W (rs755537970) variant in AIDHS. (DOCX) [file pone.0211661.s006.docx]

Table 3S-A. Demographic and clinical traits of carriers of GCKR S105N (rs774930016) variant in subjects from the AIDHS.

| Data ID | Related/ unrelated | S105N  Carrier | Sex | Age (years) | T2D | Age of T2D onset | BMI (kg/m^2^) | FTG (mg/dl) | FBG (mg/dl) |
| --- | --- | --- | --- | --- | --- | --- | --- | --- | --- |
| C0174 | Related | No | M | 45 | Yes | - | 22.2 | 205 | 136 |
| C0176 | Related | Yes | M | 42 | Yes | 42 | 32.0 | 239 | 191 |
| C0205 | Related | Yes | F | 35 | No | - | 40.6 | 182 | 109 |
| C0177 | Related | No | F | 36 | No | - | 30.1 | 167 | 106 |
| C0178 | Related | No | F | 39 | No | - | 25.5 | 94 | 98 |
| C0179 | Related | No | F | 43 | No | - | 27.3 | 109 | 89 |
| C0173 | Related | Yes | M | 43 | No | - | 19.5 | 374 | 104 |
| C0175 | Related | Yes | M | 48 | No | - | 30.8 | 192 | 102 |
| C0172 | Related | Yes | F | 65 | Yes | 58 | 26.0 | 308 | 97 |
| C0204 | Related | Yes | F | 54 | Yes | 53 | 31.0 | 438 | 131 |
| D025 | Unrelated | Yes | F | 47 | Yes | 37 | 30.7 | 184 | 209 |
| D027 | Unrelated | Yes | M | 45 | Yes | 38 | 30.3 | 122 | 219 |
| P0202 | Unrelated | Yes | F | 55 | Yes | 50 | 26.3 | 516 | 263 |

Abbreviations are as follows: T2D-type 2 diabetes, BMI-body mass index, FTG-fasting triglyceride, FBG-fasting blood glucose.

Table 3S-B. Demographic and clinical traits of carriers of GCKR R297Q (rs760427565) variant in subjects from the AIDHS.

| Data ID | R297Q Carrier | Sex | Age (years) | T2D | Age of T2D onset | BMI (kg/m^2^) | FTG (mg/dl) | FBG (mg/dl) |
| --- | --- | --- | --- | --- | --- | --- | --- | --- |
| L0212 | Yes | F | 51 | Yes | 38 | 25.7 | 530 | 237 |
| HPRK0089 | Yes | F | 50 | Yes | 45 | 31.3 | 370 | 290 |
| HCJGS0026 | Yes | M | 52 | No | - | 31.2 | 369 | 82 |
| PA0212 | Yes | F | 58 | Yes | 53 | - | 259 | - |
| HPAJK0082 | Yes | F | 62 | Yes | 49 | 24.1 | 259 | 335 |
| S006 | Yes | M | 38 | Yes | 37 | 24.4 | 190 | 145 |
| L0210 | Yes | M | 46 | Yes | 44 | 28.9 | 186 | 203 |
| S004 | Yes | F | 55 | Yes | 54 | 34.0 | 180 | 88 |
| S002 | Yes | M | 40 | Yes | 39 | 23.5 | 167 | 133 |
| P0271 | Yes | F | 35 | Yes | 34 | 25.9 | 148 | 217 |
| S025 | Yes | M | 30 | No | - | 24.6 | 122 | 107 |
| OKC348 | Yes | F | 42 | No | - | 24.5 | 114 | - |
| P0301 | Yes | F | 54 | No | - | 24.4 | - | 108 |
| PH007 | Yes | M | 56 | Yes | 36 | 32.4 | - | 133 |
| PH010 | Yes | M | - | No | - | 26.7 | - | 108 |
| CHD0345 | Yes | M | 51 | Yes | - | 26.0 | - | 200 |
| CHD0404 | Yes | M | 47 | Yes | - | 22.7 | - | 120 |
| CHD0363 | Yes | M | 59 | Yes | - | 22.5 | - | 191 |

T2D-type 2 diabetes, BMI-body mass index, FTG-fasting triglyceride, FBG-fasting blood glucose.
