# Supplementary material for: Targeted sequencing of candidate genes of dyslipidemia in Punjabi Sikhs: Population-specific rare variants in GCKR promote ectopic fat deposition
Source: PLoS One. 2019 Aug 1;14(8):e0211661. doi: 10.1371/journal.pone.0211661 (PMC6675050; doi:10.1371/journal.pone.0211661)
Supplement: S4 Table — (DOCX) [file pone.0211661.s007.docx]

Table 4S. Primer Details

| **Gene Expression Primers** | |
| --- | --- |
| hGCKR_GexpS_set2_FWD | 5’ AGGCTCGATGCATCG ‘3 |
| hGCKR_GexpS_set2_REV | 5’ CATGTGCAACCTGGAC ‘3 |
| b-actin_FWD_mRNA | 5’ AAATCGCTGCCCTGGTCGTT ‘3 |
| b-actin_REV_mRNA | 5’ CTGTCCCATGCCAACCATCA ‘3 |

| **Gene sequencing primers (Codons S105N, R297Q, R553W)** | |
| --- | --- |
| GCKR Int F | TTCCAGGAGGAGGGGCAAGC |
| GCKR Int R | CTGCTCCCATCTCCTGCCAT |

| **Details of GCKR Cloning Primers using Tol2 Technology** | |
| --- | --- |
| >attB1_primer | GGGGACAAGTTTGTACAAAAAAGCAGGCT |
| >attB1R_primer | GGGGACTGCTTTTTTGTACAAACTTG |
| >attB2_primer | GGGGACAGCTTTCTTGTACAAAGTGG |
| >attB2R_primer | GGGGACCACTTTGTACAAGAAAGCTGGGT |
| >attB3R_primer | GGGGACAACTTTGTATAATAAAGTTG |
| >attB4_primer | GGGGACAACTTTGTATAGAAAAGTTG |
| >attL1 | CAAATAATGATTTTATTTTGACTGATAGTGACCTGTTCGTTGCAACAMATTGATGAGCAATGCTTTTTTATAATGCCAACTTTGTACAAAAAAGCAGGCT |
| >attL2 | ACCCAGCTTTCTTGTACAAAGTTGGCATTATAAGAAAGCATTGCTTATCAATTTGTTGCAACGAACAGGTCACTATCAGTCAAAATAAAATCATTATTTG |
| >attL3 | CAACTTTATTATACAAAGTTGGCATTATAAAAAAGCATTGCTTATCAATTTGTTGCAACGAACAGGTCACTATCAGTCAAAATAAAATCATTATTT |
| >attL4 | AAATAATGATTTTATTTTGACTGATAGTGACCTGTTCGTTGCAACAAATTGATAAGCAATGCTTTTTTATAATGCCAACTTTGTATAGAAAAGTTG |
| >attR1 | CAAGTTTGTACAAAAAAGTTGAACGAGAAACGTAAAATGATATAAATATCAATATATTAAATTAGATTTTGCATAAAAAACAGACTACATAATACTGTAAAACACAACATATGCAGTCACTATGAATCAACTACTTAGATGGTATTAGTGACCTGTA |
| >attR2 | TACAGGTCACTAATACCATCTAAGTAGTTGRTTCATAGTGACTGCATATGTTGTGTTTTACAGTATTATGTAGTCTGTTTTTTATGCAAAATCTAATTTAATATATTGATATTTATATCATTTTACGTTTCTCGTTCAACTTTCTTGTACAAAGTGG |
| >attR3 | CCATAGTGACTGGATATGTTGTGTTTTACAGTATTATGTAGTCTGTTTTTTATGCAAAATCTAATTTAATATATTGATATTTATATCATTTTACGTTTCTCGTTCAACTTTATTATACATAGTTG |
| >attR4 | CAACTTTGTATAGAAAAGTTGAACGAGAAACGTAAAATGATATAAATATCAATATATTAAATTAGATTTTGCATAAAAAACAGACTACATAATACTGTAAAACACAACATATCCAGTCACTATGG |
